# ORCA: a comprehensive bioinformatics container environment for education and research

**DOI:** 10.1093/bioinformatics/btz278

**Published:** 2019-04-20

**Authors:** Shaun D Jackman, Tatyana Mozgacheva, Susie Chen, Brendan O’Huiginn, Lance Bailey, Inanc Birol, Steven J M Jones

**Affiliations:** Genome Sciences Centre, BC Cancer, Vancouver BC, Canada

## Abstract

**Summary:**

The ORCA bioinformatics environment is a Docker image that contains hundreds of bioinformatics tools and their dependencies. The ORCA image and accompanying server infrastructure provide a comprehensive bioinformatics environment for education and research. The ORCA environment on a server is implemented using Docker containers, but without requiring users to interact directly with Docker, suitable for novices who may not yet have familiarity with managing containers. ORCA has been used successfully to provide a private bioinformatics environment to external collaborators at a large genome institute, for teaching an undergraduate class on bioinformatics targeted at biologists, and to provide a ready-to-go bioinformatics suite for a hackathon. Using ORCA eliminates time that would be spent debugging software installation issues, so that time may be better spent on education and research.

**Availability and implementation:**

The ORCA Docker image is available at https://hub.docker.com/r/bcgsc/orca/. The source code of ORCA is available at https://github.com/bcgsc/orca under the MIT license.

## 1 Introduction

Setting up a suitable platform for effective bioinformatics analysis can be challenging. Determining the dependencies and version requirements needed to install standard software packages is a barrier before analysis can even begin. Installing a bioinformatics tool requires first installing its dependencies, which themselves have dependencies and so on. Easy-to-install software is vital to encourage reproducible data analyses (Leprevost *et al.*, 2014). Docker provides the ability to configure a computing environment that contains complex pipelines and workflows, and to distribute it across multiple platforms (Menegidio *et al.*, 2017). Several recent projects have provided bioinformatics container environments for research. For example, BioContainers gives access to bioinformatics software from the BioConda repository (Grüning *et al.*, 2018), which creates an isolated Docker container for each software package (Leprevost *et al.*, 2017), and BioBoxes serves a similar purpose (Belmann *et al.*, 2015). Combining multiple isolated containers into a single analysis pipeline may, however, be challenging for beginners, and is not suitable for a novice audience not yet familiar with containers or data analysis pipeline tools. When the goal is to teach bioinformatics, it is not desirable to first have to teach containers. A comprehensive container that provides all of the desired software in a single container is better suited in this situation to isolated containers. BioLinux (http://environmentalomics.org/bio-linux/) provides a comprehensive disk image that may be installed on a physical USB flash drive or DVD, and the related project Cloud BioLinux (Krampis *et al.*, 2012) provides an Amazon machine image for use with cloud computing services. ORCA, the Genomics Research Container Architecture, provides a comprehensive bioinformatics command line computing environment for education or research in a Docker image, which includes hundreds of popular bioinformatics tools and their dependencies.

## 2 Architecture

In an educational setting, ORCA uses Docker to provide a private containerized environment, but without requiring that the user interacts directly with Docker. The user logs into the ORCA server using the secure shell utility ssh. The user’s login shell is configured to run an interactive shell inside their private container, transparently to the user. In this configuration, the users do not themselves run or interact with Docker. The container and the users’ processes inside the container continue running when the user logs out, so that the user may later return to that same container to check status and results of their jobs. Data are transferred into and out of the container using any of the utilities scp, sftp or rsync. Graphical applications, such as the Integrative Genomics Viewer, may be used by tunneling the X11 protocol through ssh. Configuring ORCA on a multi-user server is described in the ORCA documentation (https://github.com/bcgsc/orca#readme). It uses a login shell script to present each user with a shell inside their individual container. In an individual setting, ORCA may be installed on a user’s workstation or laptop by installing Docker (https://www.docker.com/get-docker) and then running the command docker run -it -v$HOME:$HOME -w$HOME bcgsc/orca, providing access to all of the software included in the ORCA image, and mounting the user’s home directory for storage of their data. ORCA does not require physical media for installation or cloud computing, though the Docker image may be used with cloud computing if desired.

**Fig. 1.**
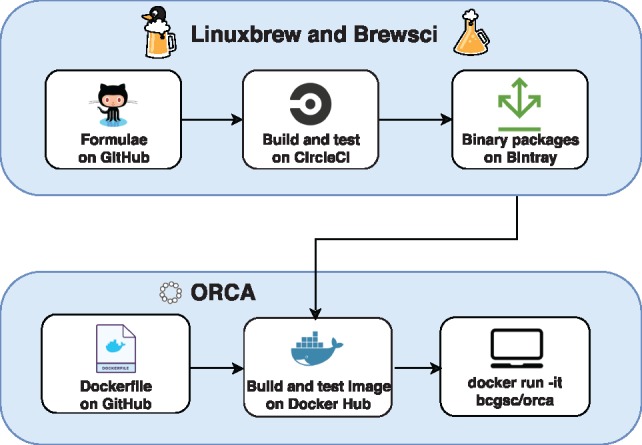
The architecture of ORCA. The package scripts of Homebrew and Brewsci, called formulae, are stored on Github. The precompiled binary packages of Homebrew are built and tested on CircleCI and stored on Bintray. The ORCA Dockerfile is stored on GitHub. The ORCA Docker image is built, tested and stored on Docker Hub. The system administrator or user pulls the image from Docker Hub to run on their server or workstation

Building the ORCA container image is automated, and an overview of the architecture is shown in Figure 1. The Dockerfile script to build the container image is stored on GitHub. The continuous integration service TravisCI is used to analyze the Dockerfile for common mistakes and pitfalls after each commit to GitHub. The container image is built by Docker Hub from the Dockerfile stored on GitHub using the automated builds feature of Docker Hub. Tagging a new release of ORCA on GitHub causes a new Docker image to be built, tested and tagged on Docker Hub. Each stable release of the ORCA image is assigned a version number, so that users may pull an earlier version of ORCA from Docker Hub to repeat a previous analysis. Automating the build and test procedure of the ORCA image permits deploying a new image in half a day and ensures a reproducible build of the Docker image. Although the Docker image is large (17 GB), it is smaller than a single lane of DNA-sequencing data (more than 50 GB).

The majority of the software packages distributed with ORCA are installed using the Homebrew (https://brew.sh) package manager. Homebrew can install software in the user’s home directory on Linux, macOS or Windows, using Windows Subsystem for Linux, and does not require administrator privileges (Jackman and Birol, 2016). Homebrew provides precompiled binaries for many packages, which alleviates the need to build each tool from source. A binary package is built on CircleCI and stored on Bintray whenever each tool is updated. Building the ORCA Docker image would take many days if each package were built from source when the image is built.

The bioinformatics packages available to Homebrew are pre-installed in ORCA. Users may install other packages that are not included by default with ORCA. The Debian package management tool apt-get and the language-specific package management systems for Perl (cpan), Python (pip) and R may be used to install additional software packages. Interested and engaged users may contribute new bioinformatics tools, or new versions of existing tools, to Brewsci/bio (https://github.com/brewsci/homebrew-bio), which builds binary packages for both Linux and macOS. These contributed tools will be included in the next release of ORCA.

## 3 Example uses of ORCA

The Genome Sciences Centre of BC Cancer uses ORCA to provide access to their compute resources to external collaborators. Giving collaborators direct access to the internal cluster could risk exposing sensitive patient data. Each collaborator is instead provided with a private container, which also isolates their data from the view of other collaborators.

A 4-month undergraduate microbiology class at the University of British Columbia uses ORCA to teach the concepts and applications of bioinformatics research to biologists. The 85 students in 2018 each had their own isolated container, protecting their individual work from each other, and access to a shared course folder provided by the instructor.

Hackseq (https://hackseq.com) is a Vancouver-based 3-day hackathon focused on genomics that brings individuals with diverse backgrounds together to collaborate on scientific questions and problems in genomics (hackseq Organizing Committee, 2016). In a hackathon environment, installing and configuring software can consume much of the limited time available for the event. ORCA provided a ready-to-go bioinformatics environment for the 98 participants in 8 teams of Hackseq in 2018. The members of a team each have their own user account and home directory, access to a shared project folder and share a single container. Sharing a single container allows system resources like memory (RAM) to be allocated to and shared by the team as a whole. ORCA allows participants to spend less time installing tools and more time developing their project.

These projects used two Dell FC630 servers. Each server is equipped with two Intel Xeon E5-2650 2.3 GHz CPUs (40 CPU cores per server), 128 GB of RAM and 5 TB of shared network storage.

## 4 Conclusions

ORCA provides a comprehensive bioinformatics container environment, which may be installed with a single Docker command, and includes hundreds of pre-compiled and configured bioinformatics tools. It may be used to painlessly install a multitude of bioinformatics tools on a fresh Linux server, provide a private container to each individual user, or shared containers to a collaborative group of users. It has been used successfully to provide compute resources to external collaborators at a core sequencing facility, to introduce bioinformatics to undergraduate biology students, and to provide a ready-to-go bioinformatics environment for a collaborative hackathon.
